# Transcriptomic responses to location learning by honeybee dancers are partly mirrored in the brains of dance-followers

**DOI:** 10.1098/rspb.2023.2274

**Published:** 2023-12-20

**Authors:** Fabio Manfredini, Yannick Wurm, Seirian Sumner, Ellouise Leadbeater

**Affiliations:** ^1^ Present address: School of Biological Sciences, University of Aberdeen, AB24 3UL Aberdeen, UK; ^2^ Department of Biological Sciences, Royal Holloway University of London, TW20 OEX Egham, UK; ^3^ School of Biological & Behavioural Sciences, Queen Mary University of London, E1 4NS London, UK; ^4^ Digital Environment Research Institute, Queen Mary University of London, E1 4NS London, UK; ^5^ Department of Genetics, Evolution and Environment, University College London, WC1E 6BT London, UK

**Keywords:** social learning, neurogenomics, gene expression, waggle dance, distance, direction

## Abstract

The waggle dances of honeybees are a strikingly complex form of animal communication that underlie the collective foraging behaviour of colonies. The mechanisms by which bees assess the locations of forage sites that they have visited for representation on the dancefloor are now well-understood, but few studies have considered the remarkable backward translation of such information into flight vectors by dance-followers. Here, we explore whether the gene expression patterns that are induced through individual learning about foraging locations are mirrored when bees learn about those same locations from their nest-mates. We first confirmed that the mushroom bodies of honeybee dancers show a specific transcriptomic response to learning about distance, and then showed that approximately 5% of those genes were also differentially expressed by bees that follow dances for the same foraging sites, but had never visited them. A subset of these genes were also differentially expressed when we manipulated distance perception through an optic flow paradigm, and responses to learning about target direction were also in part mirrored in the brains of dance followers. Our findings show a molecular footprint of the transfer of learnt information from one animal to another through this extraordinary communication system, highlighting the dynamic role of the genome in mediating even very short-term behavioural changes.

## 1. Background

Honeybee colony foraging is a paradigm of self-organization, driven by a series of signals that regulate the shifting distribution of foragers between sites through a number of feedback loops [1–5]. Central to this system is the waggle dance, through which foragers indicate the location of resources that they have found. Within each waggle dance, the duration of the central run predicts distance to the resource, while its orientation indicates direction in the field relative to the sun's azimuth [4]. In most cases, foragers that follow waggle runs already know of the resource indicated in the dance, and following it may serve only to trigger navigational memories or provide information about current productivity [6–8]. However, visits to unfamiliar resources are typically preceded by multiple bouts of dance-following that increase in intensity over time, over which followers learn the spatial location of the new site [9]. As a result, the vast majority of arrivals at a new site are driven by learning of navigational information within the darkness of the hive, in the absence of spatial stimuli [8]. As yet, the mechanisms by which this is achieved have received little research attention (but see [10,11]).

Behaviour is regulated by the brain, and it is now apparent that even transient behavioural states can be accompanied by distinct gene expression profiles in the neural tissue (reviewed in [12–14]) from fish [15,16], to birds [17] and insects [18–21]. In honeybees, a series of studies have linked distinct neural gene expression changes to key behavioural patterns, such as, for example, scouting for food [22–24], orientation flights [25], and responses to food type, reward and value [26]. Intriguingly, these findings extend to small differences in neural gene expression that correspond with the location of the feeder that a bee has visited [27], and specifically the distance that a bee perceives herself to have flown. Using a classic protocol to manipulate optic flow [28,29], Sen Sarma *et al.* found that bees perceiving themselves to have flown short or long distances differed in their neural gene expression profiles, despite having covered the same distance [27]. Such findings raise the question of whether following dances can induce similar differential patterns in dance-followers, who must reverse-engineer a flight vector from the orientation and duration of the central waggle run of each dance that is followed.

In this study, we set out to characterize the neural transcriptomic profiles of dance followers as they learnt about foraging locations from within the darkness of the hive, through transcriptomic analysis of the mushroom bodies—one of the main structural regions of the insect brain responsible for integration of a wide range of information from different sources for learning and memory [30,31], including within spatial learning paradigms [32,33]. Based on previous research [27], we hypothesized that the global neural gene expression patterns of dancing bees would differ according to the distance and/or direction of the forage site that they had visited, and critically, that aspects of such differential patterns may also be detectable in the brains of bees that followed such dances.

## 2. Methods

### (a) Subjects

Observation hives were installed between May and July in two consecutive years (2016 and 2017), sourced from full honeybee colonies (British commercial honeybees, *Apis mellifera*, Paynes Southdown Bee Farms) situated in the university apiary. Hives were housed inside at 26°C and 40% humidity in dark conditions. At initiation, each hive contained a functional egg-laying queen (Buckfast mated queen), two frames of brood of mixed age (approximately 3000 workers) and a small frame containing food stores (mainly nectar and some pollen). Clear Plexiglas walls enabled us to look inside the hives from both sides. Hives were connected through a clear pipe to the outside, so that honeybees could freely forage in the outer environment. Hives were installed a minimum of two weeks before we started the experiment.

### (b) Obtaining cohorts of dancers and followers for feeders located at different distances and directions from the hive

To create groups of bees that were motivated to follow dances for new feeders, we trained two cohorts of bees in each trial. On the first day of the experiment, we trained a cohort of foragers from a focal hive to a feeder (‘F1’) containing a 2 M sucrose solution, which was slowly moved to the final location (165 m eastward; training took 2 days). Each bee was marked with a unique combination of three coloured dots (Enamel) on their thorax, and any bees at the feeder that were not observed in the focal hive during the experiment were removed. An observer continually monitored the feeder to record arrivals. Once this cohort was trained to the final target location, we started to train a different group of bees from the same colony to visit another feeder (‘F2’), using the same methods, placed at a different final location. An observer was always present, so we can be sure that bees from F1 did not visit F2. For distance experiments, F2's final location was at 200 m from the hive for the LONG treatment, and at 35 m for the SHORT one, always to the South of the hive. In the direction experiment, F2's final location was 200 m NORTH or SOUTH, depending on the treatment. Again, all bees were marked.

On the morning of day 5 of the experiment, we activated only F2 while we left F1 empty. F1 foragers would initially visit their feeder as usual, but then, on finding it empty, would start to follow dances for F2. We positioned observers at F1, F2 and the focal hive for the whole duration of the experiment. We also positioned a camera in front of the hive and filmed the dancefloor for the 2 h preceding sampling (approx. 10.00–12.00). At noon, we sampled all F1 foragers that did not reach F2, at the entrance of the hive as they left the colony to engage in the next foraging trip. By analysing the videos preceding this collection, we could ascertain whether each bee had followed dances for F2, and if so, how many circuits had been followed: these bees were called followers. We also sampled any F1 forager that reached the F2 feeder at their first successful attempt (called recruits; for analyses of this group see electronic supplementary material). Finally, we sampled F2 foragers both at the feeder and immediately after they left the hive: these are dancers. Bees were first housed in a container in the dark for 20 min, to allow for the time course of early gene expression following exposure to a stimulus [34] (although note that the time since exposure was likely considerably longer, because bees followed dances throughout the two hours of the experiment duration). Thereafter, they were flash frozen in liquid nitrogen and subsequently stored in a −80°C freezer for later processing for molecular work.

We repeated this experimental protocol every week from the beginning of August to October, using one of three colonies at a time and alternating between long-distance versus short-distance, and North versus South treatments, so that each week included more than one treatment and each treatment was repeated across the whole duration of the experimental period. At the end of the season, we had 17 trials with multiple replicates for all bee groups from each of the three colonies and for all four treatments (see electronic supplementary material).

### (c) Manipulating distance perception using patterned tunnels

In a separate experiment, following Srinivasan *et al*. [35] and Sen Sarma *et al*. [27] (see also [36,37]), we used a patterned wooden tunnel (0.2 × 0.2 × 6 m) to manipulate optic flow and thus honeybee perception of distance flown. The far end of the tunnel was closed, therefore foraging bees could enter the tunnel only through the end next to the colony entrance, and the top of the tunnel was covered with a metal mesh so that bees had a view of the sky to orient their flights. The entrance of the tunnel was in an external unshaded area approximately 7 m away from the entrance pipe that led to the observation hive. For the ‘long distance’ treatment, the lateral interior walls of the tunnel contained alternating black and white *vertical* stripes, to induce relatively high optic flow and thus perception of longer-distance flight (approx. 200 m). For the ‘short distance’ treatment, the same stripes were aligned horizontally, inducing relatively less optic flow. This set-up has been shown to successfully manipulate distance perception in past research [35–37], including to investigate differences in gene expression [27]. Correspondingly, when we analysed the dances of bees that encountered the two treatments, we found that bees in the long-distance treatment performed dances with a significantly higher waggle ratio, as is expected for bees perceiving longer distances (data not shown; see also [27]).

We trained bees from the focal hive (*n* = 4 colonies used in succession) to visit a feeder located at the closed end of the tunnel exactly in the same way as described above, except that the feeder was moved in steps of approximately 20 cm through the tunnel. Training took approximately 5 h. Once the feeder was at its target location, we videoed the dancefloor for an interval of 2 h. An observer took note of all marked bees performing dances and also all marked bees that were visible on the dancefloor but were not dancing. At the end of the 2 h interval (around 16.00) we sampled all marked bees at the feeder and we flash froze them in liquid nitrogen. Thereafter, bee samples were stored in a −80°C freezer for later processing for molecular work.

We repeated the procedure described above on a daily basis, from the end of July to the end of September, alternating the two treatments (vertical stripes and horizontal stripes) for four focal hives, until we had samples for each treatment by colony combination. We used 32 bee samples for the RNAseq experiment, representing the four focal hives across the four experimental groups: eight dancers that perceived long distance + 8 dancers that instead perceived short distance (DDL and DDS groups, respectively); we also added two additional groups composed of bees from the same cohorts as above, hence exposed to the same treatments, that were never seen dancing in the 2 h interval before collection (non-dancers for long and short treatments, NDL and NDS, respectively; see [38] for analyses involving these bees).

### (d) Preparation of samples for molecular work and RNA sequencing

We used video analysis of waggle dances performed during our trials to select samples for RNAseq analysis. In our tunnel experiments, we were careful to ensure that the foragers we selected for sequencing had perceived the distance flown as intended by our manipulation; thus we selected dancers with >30% waggle ratio for the long-distance treatment, and dancers with <10% waggle ratio for the short-distance treatment. Waggle ratio was calculated as follows. Videos were observed for the whole 2 h period preceding collection of the samples and all dance events were analysed. Each dance event contained one or multiple circuits, composed by the central waggle run and a hemicycle to the right or to the left that the bee used to resume her position. We counted all circuits when a bee shook her abdomen during the waggle run and we divided them by the total number of circuits of that specific dance event. We then averaged the obtained ratio across all dance events observed for the same bee within the 2 h period. For all the other groups, we checked that all bees in ‘dancer’ samples were indeed dancing for the appropriate feeder before they left the colony for the last time, and that ‘followers’ had been following such dances. We also recorded the number of dance circuits followed immediately before a follower left the colony for the final time.

Mushroom bodies from each bee destined for RNAseq analysis were dissected out on dry ice under a stereomicroscope, immediately immersed in 450 µl QIAzol Lysis reagent (Qiagen, Manchester, UK) and homogenized in a Tissue Lyser device (Qiagen). Total RNA was isolated from individual mushroom bodies samples with the RNeasy Plus Universal kit (Qiagen) following manufacturer instructions. RNA quantities and quality were first checked on site with a NanoDrop spectrophotometer instrument (ThermoFisher), and then subsequently validated on an Agilent Bioanalyzer at the sequencing facility (GENEWIZ NGS Lab, South Plainfield, NJ, USA), where cDNA synthesis, library preparation and sequencing on an Illumina HiSeq 2500 platform (2 × 150 bp configuration) took place.

Library preparation was obtained with the strand-specific polyA selection kit for all followers and dancers included in the main experiment (testing the response to variation in distance and direction). These samples were run in two separate batches: followers were run on 12 lanes and dancers were run on 7 lanes of the sequencer (on average, 41.18 and 42.42 million raw paired-end reads per sample, respectively). Note that all samples for direct comparison (e.g. followers for LONG versus SHORT feeder) were run in the same batch. Foragers sampled from the tunnel paradigm, carried out in a different year, were processed with the Strand-specific rRNA depletion kit and all 32 samples were run on 4 lanes (43.75 million reads per sample, on average). In all cases, libraries were individually indexed and multiplexed on each lane of the sequencer, so that all lanes contained a range of representatives from all relevant treatments and colonies.

### (e) Analysis of gene expression

RNAseq read files were aligned to the most recent version of the *A. mellifera* genome (Amel_4.5) using the intron-aware STAR aligner, version 2.6.1a [39]. Read counts were extracted using *featureCounts,* part of the Bioconductor R package Subread, version 1.8.0 [40]. We generated a list of raw numbers of reads per gene. Raw reads were normalized for sequencing depth and this output was used to calculate differential gene expression using the R package edgeR following two approaches: (a) planned pairwise comparisons between groups of interest among dancers and followers tested for different perception of distance and direction, and (b) a glmLRT approach for follow-up analyses of gene expression to compare followers versus non-followers, above versus below threshold of circuit following and followers versus recruits (see electronic supplementary material). All analyses of gene expression were performed within batch, to avoid any possible confounding effect deriving from the allocation of each sample to a specific batch of sequencing. This means that the effect of distance was assessed in dancers separately from followers, and the same for direction: common elements between list of differentially expressed genes were then identified by means of overlap analysis. Differential expression was invoked for *p*-values < 0.05 after correction for multiple testing (false-discovery rate or FDR). Overlap analysis were performed with Venny (https://bioinfogp.cnb.csic.es/tools/venny/index.html) and we used a Hypergeometric test to evaluate whether the overlap was significantly larger than expected by chance. We also performed additional analyses with the R package DESeq2 to investigate the possibility that the colony of origin could influence gene expression. For this purpose, we used a likelihood ratio test (LRT) where we compared the output of a full model including treatment (distance or direction perceived) and colony of origin versus the reduced model that included colony alone. This set of analyses revealed a pool of 179 genes that were influenced by colony of origin in dancers overall (FDR < 0.05), while no genes were detected in followers. There was no overlap between these genes and the list of best candidates for distance and direction perception (see Results). Network analyses were performed with the R package WGCNA (weighed gene co-expression network analysis [41]) and are fully described in the electronic supplementary material.

## 3. Results

### (a) Learning about distance

**Figure 1.**
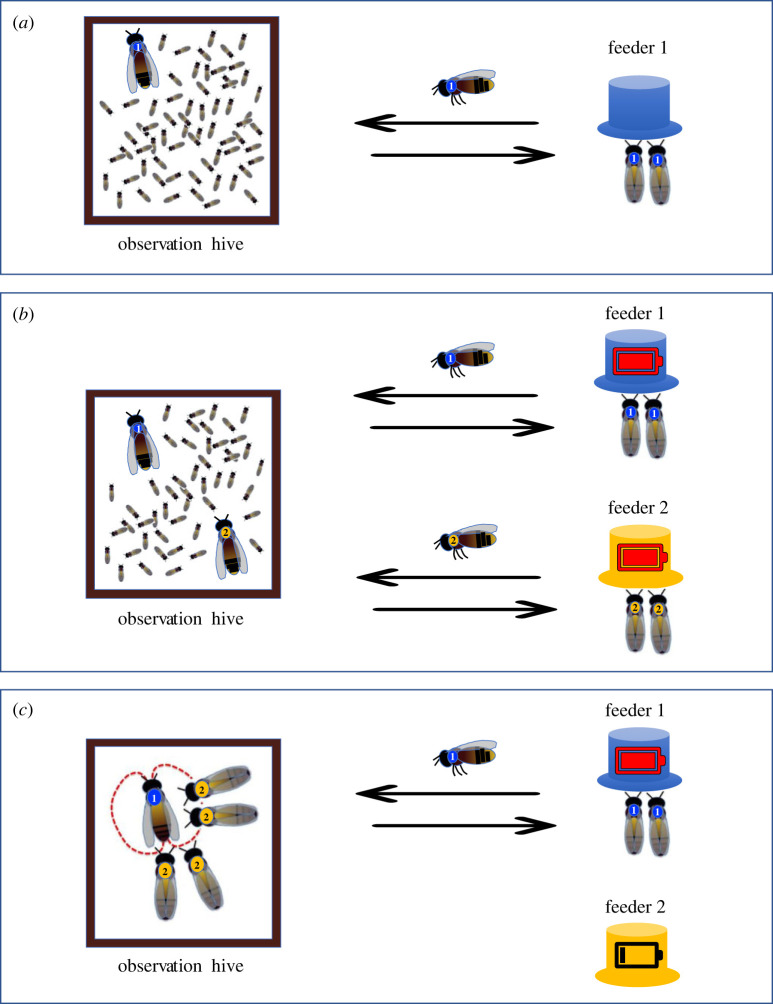
Experimental set-up to obtain honeybee followers: (*a*) a first cohort of bees is trained to a feeder (feeder 1) containing a sucrose solution; (*b*) a second cohort of bees is trained to a second feeder (feeder 2) located in a different location compared to feeder 1; (*c*) feeder 2 is left empty so that foragers that were regularly visiting this feeder are temporarily unemployed and will start following dances for feeder 1.

Over six paired trials, carried out on three colonies in observation hives in succession, we trained cohorts of foragers to visit feeders located either 200 m (‘LONG’ trials) or 35 m (‘SHORT’ trials) from the hive (figure 1*a,b*). Each trial involved only one type of feeder and colony, and all colonies participated in both one LONG and one SHORT trial. We caught a subsample of each cohort (dancers) for subsequent RNAseq analysis of the mushroom bodies. At the same time, we sampled foragers from the same colonies that attended dances but had not yet visited the target feeder. These ‘followers’ were individually marked temporarily unemployed foragers that had been previously visiting a different feeder which had become unrewarding (figure 1*c*). We recorded all dance interactions in the hive, and followers were caught and flash-frozen immediately on leaving the hive, just after following a dance for one of the two feeders.

We found several lines of evidence to confirm that a set of genes responded to the distance to the feeder that a bee had visited and danced for, and that some of these patterns were also present in the bees that followed these dances to learn about new forage sites. We started with the transcriptomic analysis of dancers, each of which had consistently visited the focal feeder over the entire training period and danced for it in the hive repeatedly on the day of sampling (LONG, *n* = 18, and SHORT, *n* = 17). This analysis revealed that 874 genes were significantly differentially expressed between the LONG and SHORT dancer groups (*p*-value < 0.05), but this number was visibly reduced to 141 genes after correcting for multiple testing (FDR < 0.05). Interestingly, 57% of the detected genes were more highly expressed in dancers for the long-distance feeder (see electronic supplementary material, for a list of these genes). We then analysed global gene expression in the mushroom bodies of the two groups of followers of the same dances, none of which had previously visited the target sites. We found that 653 genes were significantly differentially expressed between the LONG and SHORT groups (*n* = 18 and *n* = 7, respectively, *p*-value < 0.05) but only 76 remained following correction for multiple testing (FDR < 0.05), with 64% of them being more highly expressed in followers for long distance (see electronic supplementary material, for a list of these genes). Thus, we found evidence for transcriptomic responses to LONG versus SHORT distance in both the dancer group and the follower group.

**Figure 2.**
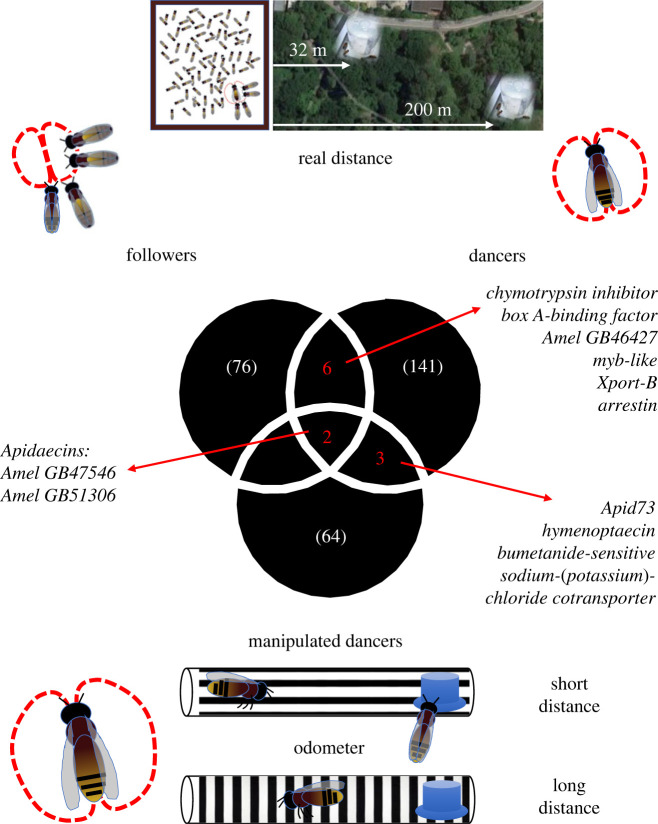
Genes responding to learning about distance. Overlap analyses of set of genes that were differentially expressed after pairwise comparisons between three groups of bees: (1) dance followers; (2) dancers for real distance; (3) dancers for perceived distance. Top: locations of the long-distance and short-distance feeders. Bottom: design of the odometer to manipulate distance perception in honeybee foragers.

To establish whether the genes that responded to distance learning in followers were similar to those that responded in dancers, we overlapped the two lists of differentially expressed genes. We found that eight genes were in common (figure 2): this represents a significantly larger overlap than expected by chance (Hypergeometric Test, Representation factor: 11.4, *p*-value < 10^−6^). In other words, these genes were differentially expressed between both dancers for LONG and SHORT distance, and followers for LONG and SHORT distance. The shared genes included *box A-binding factor* (GB50932), a transcriptional regulatory factor [42] and 2 *apidaecins* (GB47546 and GB51306). *Apidaecins* are taxonomically restricted genes that appear to be rather honeybee-specific [43], although apidaecin-like peptides are found in other insects, mainly within the Hymenoptera (see Discussion for description of their known function). *Apidaecins* were also over-represented in the set of candidate distance-responsive genes described by Sen Sarma *et al.* [27], constituting 13% of the differentially expressed set in that study. Both *apidaecins* and *box A-binding factor* were more highly expressed in both dancers and followers for short distance. The other genes in the overlapping set had opposite patterns for the two analyses (figure 2), so we do not consider these likely candidates for responses to distance perception.

To recap, we performed a third experiment to independently verify the potential role of such genes in learning of the distance to the target. Following Sen Sarma *et al.* [27], we used a standard tunnel-based optical flow paradigm [35,44] to compare foragers that visited two feeders located at the same distance from the hive while perceiving different distances (*n* = 8 per group). Sampled bees were from 8 paired trials (4 LONG, 4 SHORT) carried out on four different colonies (year of trial and colonies differed from those reported above) and had performed repeated dances on the day of collection that advertised the distances to which they had been trained (see electronic supplementary material). We found that 835 were significantly differentially expressed (*p*-value < 0.05) between dancers that perceived long distance versus short distance, but only 64 genes remained after correcting for multiple testing (FDR < 0.05, see electronic supplementary material, for a list of these genes and details on their functions), most of them being more highly expressed for short distance (72%). Again, we carried out overlap analyses to establish whether this set of genes mirrored our findings in dancers for LONG versus SHORT distance: five genes were in common with differentially expressed genes in dancers for real distance (figure 2), representing a significant overlap between the two sets (Hypergeometric Test, Representation factor: 8.5, *p*-value < 10^−3^). The shared genes were 3 *apidaecins* (GB46236, GB47546 and GB51306), *hymenoptaecin* (GB51223) another Hymenoptera-restricted gene known for its role in antibacterial response [45] and *bumetanide-sensitive sodium-(potassium)-chloride cotransporter* (GB40283), a member of the NKCC family known for its role in visual synaptic transmission in *Drosophila* [46]. With the exception of the last one, all shared genes were consistently expressed at higher levels in dancers for short distance in both analyses. Only two of these genes were also differentially expressed in followers that learned about different distance: these were the two *apidaecins* GB47546 and GB51306, again more highly expressed for short distance. These two *apidaecins* therefore responded consistently and in the same direction to distance perception across all three types of comparison: LONG versus SHORT dancers for real distance, LONG versus SHORT dancers for perceived distance, and LONG versus SHORT dance followers.

### (b) Learning about direction

**Figure 3.**
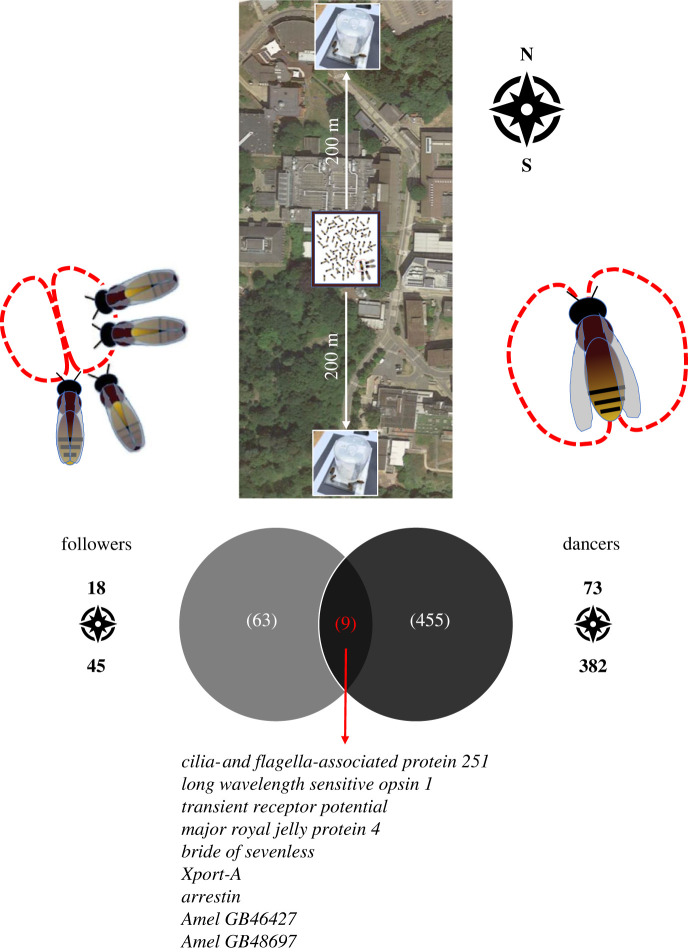
Genes responding to learning about direction. Overlap analyses of set of genes that were differentially expressed after pairwise comparisons between two groups of bees: (1) dance followers; (2) dancers. Top: locations of the NORTH and SOUTH feeders.

In a second set of assays, we analysed brain gene expression associated with direction in followers and dancers using a similar setup as described for distance. This time, however, the two alternative target feeders were positioned at equal distance to the hive (200 m) but in opposite directions, i.e. NORTH and SOUTH of the hive (figure 3).

We first analysed gene expression in dancers that regularly visited the two feeders and were seen dancing for them (*n* = 20 for the NORTH feeder and *n* = 18 for the SOUTH feeder). This comparison revealed that 1722 genes were significantly differentially expressed (*p*-value < 0.05), but this number was reduced to 445 after correcting for multiple testing **(**FDR < 0.05, see electronic supplementary material, for a list of these genes): 84% of these genes were more highly expressed in dancers for the SOUTH feeder. We then analysed brain gene expression in followers that were sampled just after following a dance for the NORTH or the SOUTH feeder (*n* = 19 and *n* = 18, respectively). The comparison between the two groups revealed that 572 genes were differentially expressed (*p*-value < 0.05), resulting in a much smaller set of 63 after correcting for multiple testing (FDR < 0.05, see electronic supplementary material, for a list of these genes). Again, a large majority of these genes (71%) were more highly expressed in followers for the SOUTH feeder. An overlap analysis between followers and dancers for NORTH versus SOUTH feeders showed that nine genes were in common between the two sets of genes (figure 3): this is more than expected by chance (Hypergeometric Test, Representation factor = 4.8, *p-*value < 10^−4^). The *apidaecins* described above did not feature in that set, as expected if they respond specifically to distance. Shared genes included: *arrestin* (GB51068), which also featured in our distance-responsive set for followers and for dancers (but not in the optic flow manipulation set), *bride of sevenless* (GB49477) with a potential role in neural regulation of photoreception [47], *cilia- and flagella*-*associated protein 251* (GB40719), *E3 ubiquitin-protein ligase protein PFF1365c* or *Xport-A* (GB50478), *long wavelength sensitive opsin 1* (GB50196), *major royal jelly protein 4* (GB55206), *transient receptor potential* (GB41297) and two genes with unknown function (GB41777 and GB48697).

## 4. Discussion

It has long been recognized that at least some forms of learning are implemented at the level of the genome, and specifically that formation of long-term memories in insects requires transcription [48,49]. Here, rather than focussing on the general process of learning by comparing groups that do or do not learn [50], we compared groups that acquire different forms of learnt information. We found—in line with previous results described by Sen Sarma and colleagues [27]—that a small group of differentially expressed genes characterized groups of bees that had directly visited either close or distant foraging sites. Subsets of these genes were also differentially expressed in bees that followed dances for those close or distant sites, and thus learnt about their locations, and in bees that had been manipulated to perceive themselves as flying short or long distances but in reality had not done so. We accepted as candidates for genes that may play a role in learning about feeder locations only those genes that were present across all three contexts. We are confident that these patterns cannot be explained by sequencing batch, as all analyses of differential expression were performed within batch, nor by effects that were specific to the colony of origin (as validated by our LRT model; see Methods section).

We found several genes that met these criteria, but the most promising candidates were two *apidaecins* that consistently reflected learning about distance to a feeder in dancers, followers, manipulated dancers and in a previous study examining a similar question. *Apidaecins*, first described in 1989 [45], have traditionally been associated with antimicrobial function (e.g. bacterial clearance) as they code for a group of four small proline-rich antimicrobial peptides [51]. However, a recent body of research has focussed on understanding the role of antimicrobial peptides beyond their immune function, particularly in association with their detection in key tissues such as the brain [52]. In fact, the commonalities in the structure of antimicrobial peptides and neuropeptides strongly suggests that these molecules could have a role in the normal functioning of the nervous system [53,54]. Intriguingly, a recent study in *Drosophila* has shown that an antimicrobial peptide (*diptericin*) is required for the formation of long-term memory associated with foraging behaviour [55]. Similar studies are not yet available for honeybees, but some preliminary observations have reported that apidaecin is detectable in the brain by mass spectrometry [56] and also that this peptide is more abundant in the brain of forager bees compared to nurses [57]. Therefore, there is suggestive evidence that points at a role for *apidaecin* in the regulation of cognitive functions in the honeybee mushroom bodies associated with foraging behaviour. Given that our series of experiments were repeated across colonies, balanced within colonies, and carried out in different years, the two *apidaecins* that we detected represent the strongest candidates for distance perception and learning that arise from our dataset.

With regards to learning about direction, our dataset again identified a set of genes that were differentially expressed between bees that flew south (towards the sun's azimuth around midday in the Northern Hemisphere) or north (away from it), and a small subset of these genes were also differentially expressed by bees that followed north-directing versus south-directing dances without visiting the feeders. Since we could not include a manipulation of perceived direction, we are cautious in our interpretation of this overlapping set. Several of the shared elements were genes that are known for their role in the perception of visual stimuli (e.g. *arrestin*, *bride of sevenless*, *Xport-A*, *long wavelength sensitive opsin 1* and *transient receptor potential*). Honeybees are not unique in that they use visual perception of the projection of the sun on the horizon to estimate the direction of their flight path, such that dancers in our SOUTH treatments flew towards the sun, and NORTH bees flew away from it. Many organisms use the sun to direct their daily navigational movements [58] and also big migratory events (e.g. monarch butterflies [59]). Interestingly, a recent study on black cutworm moths has revealed that larger proportions of genes are expressed at higher levels when these insects migrate southward compared to northward migrations [60]. However, within the context of our study it is not clear why such genes should be activated in followers, who decode dances in the dark, and in contrast to the distance set, none of the identified candidates have been previously associated with cognitive function.

It is clear that only a small portion of the information that a foraging animal learns is ever likely to induce a transcriptomic response. Across the insects, short- and mid-term memories that are retained on the scale of minutes to hours are not affected by transcription inhibitors, and are instead driven by synaptic signalling cascades that are initiated upon experiencing a single pairing between a stimulus and reward. Only formation of long-term memory, typically elicited by experiencing repeated pairings between stimuli [61,62] (but see [63,64]) requires transcription, which in *Apis* is accompanied by changes in the density of synaptic complexes within the mushroom bodies [50,65]. Accordingly, information regarding the location of food sources is relevant over several days for honeybees, which are remarkably persistent in visiting discovered food sources and return repeatedly after overnight pauses and extensive spells of bad weather, even long after the target sites have ceased to provide rewards [8,66,67]. The finding that such responses are mirrored in dance followers invites empirical testing of the intriguing possibility that waggle dance communication induces site-specific memories that may be long-lasting and stored in the long-term memory of dance followers, even before the target sites are visited.

Waggle dance communication involves an extraordinary form of social learning that takes place in the darkness of the hive, through an abstract symbolic representation of a stimulus, and our study identified elements of mirrored gene expression between dancers and followers elicited by that process. While limited clear parallels of dance communication exist outside of *Apis,* synchronized brain gene expression between interacting individuals nonetheless has been previously described in other contexts. For example, the neural transcriptomes of fighting fish *Betta splendens* become more similar over the course of an aggressive encounter, particularly with regard to genes associated with learning and memory [16]. This suggests that the interacting fish come to achieve a similar neurophysiological state through a mutual assessment process. More generally, social learning through observation (i.e. without direct reward or punishment) has been found to elicit transcriptomic responses, such that watching aggressive confrontations between conspecifics has been shown to correlate with expression of genes associated with neuronal plasticity and memory formation in ‘eavesdropping’ zebrafish *Danio rerio* [68] and social learning of fear responses elicits *c-fos* expression in distinct neural circuits to individual learning in the same species [69]. These studies differ from the current work in that the focus is not on the learnt information itself, but they illustrate that process of learning from another animal appears to be detectable in the transcriptome.

Our study was exploratory by nature; we set out to confirm whether learning about location of a food site elicits a transcriptomic response, and whether that response is mirrored in individuals that learn the same information through a very different mechanism. Our findings raise the intriguing possibility of cognitive responses to ecologically important stimuli at the level of the genome, inviting further exploration and manipulation of the candidate genes that we identified.

## Data Availability

All transcriptomic data are deposited in the NCBI SRA database under three separate BioProjects: PRJNA760896 for real-distance dancers, PRJNA762185 for real-distance followers and PRJNA756776 for the samples that were part of the tunnel simulation of foraging distance; this set also including data that were part of another publication [56]. All these data are publicly available. Supplementary material is available online [70].
